# Cancer incidence estimates and mortality for the top five cancer in Colombia, 2007-2011

**DOI:** 10.25100/cm.v49i1.3596

**Published:** 2018-03-30

**Authors:** Constanza Pardo, Ricardo Cendales

**Affiliations:** 1 Grupo de Vigilancia Epidemiológica del Cáncer, Instituto Nacional de Cancerología, Bogotá, D.C., Colombia.

**Keywords:** incidence, mortality, cancer, epidemiology, Colombia, incidencia, mortalidad, cáncer, epidemiología, Colombia

## Abstract

**Objectives:**

To describe the incidence and mortality for the five main types of cancer in Colombia, from 2007-2011.

**Methods:**

We estimated cases and cancer incidence rates standardised by age, based on incidence/mortality ratios; and we calculated the observed deaths and mortality rates standardised by age in Colombia, both differentiated by province, type of cancer and sex. Incidence estimates were generated based on information from four cancer population registries (Cali, Pasto, Bucaramanga and Manizales), published in *Cancer Incidence in Five Continents*, volume X, and the official mortality and population information of the National Administrative Province of Statistics (DANE, for its initials in Spanish).

**Results:**

The annual number of expected cases (all cancers) was 62,818 in men and women; and there were 32,653 recorded deaths. The main incidental cancers were prostate (46.5 per 100,000 person-years) in men, and breast (33.8 per 100,000 person-years) in women. The highest mortality figures were for stomach cancer in men (14.2); and breast cancer in women (9.9).

**Conclusions:**

The highest incidence and mortality estimates in Colombia were for breast and prostate cancers, as well as a proportion of infection-related cancers, such as stomach and cervical cancer. These four neoplasms were responsible for more than 50% of the burden of the disease. Only through good quality, long-duration cancer registries, can information be obtained about the changes in incidence trends.

## Resumen

**Objetivos:**

Describir la incidencia y mortalidad para los cinco principales tipos de cáncer en Colombia, de 2007-2011.

**Métodos:**

Se estimaron casos y tasas de incidencia de cáncer ajustadas por edad a partir de razones incidencia/mortalidad y se calcularon las muertes observadas y tasas de mortalidad ajustadas por edad en Colombia, ambas diferenciadas por departamentos, tipo de cáncer y sexo. Las estimaciones de incidencia se generaron con base en la información de cuatro registros poblacionales de cáncer (Cali, Pasto, Bucaramanga y Manizales), publicada en *Cancer Incidence in Five Continents*, volumen X, y la información oficial de mortalidad y población del Departamento Administrativo Nacional de Estadística (DANE).

**Resultados:**

El número anual de casos esperados (todos los cánceres) fue 62,818 en hombres y en mujeres y se registraron 32,653 muertes. Los principales cánceres incidentes fueron próstata (46.5 por 100,000 años-persona) en hombres y mama (33.8 por 100,000 años-persona) en mujeres. La mortalidad más alta en hombres se presentó en estómago (14.2) y mama en mujeres (9.9).

**Conclusiones:**

Las estimaciones de incidencia y mortalidad más altas en Colombia fueron para los cánceres de mama y próstata, además de una proporción de cánceres relacionados con la infección como son el cáncer de estómago y de cuello uterino. Estas cuatro neoplasias fueron responsables de más del 50% de la carga de la enfermedad. Solamente a través de los registros de cáncer de buena calidad y de larga trayectoria podrá tenerse información sobre el cambio en las tendencias de incidencia**.**

## Introduction

Colombia is considered to be a country with an intermediate incidence of cancer within the world panorama, with rates standardised by age estimated by the International Agency for Research on Cancer (IARC) of 175.2 cases per 100,000 men, and 151.5 cases per 100,000 women, excluding non-melanoma skin tumors ^1^. For cancer, there are established methods to measure the incidence and mortality by location at the population level: the population-based cancer registries (RCBP, for its initials in Spanish), which collect information on the incidence and the vital statistics systems of the countries that provide mortality data ^2^.

Achieving national coverage through a group of cancer registries generates a high cost ^3^; therefore, a viable alternative is to use information from a few RCBP located in strategic areas and the mortality data available at national level. The purpose is to obtain reliable national estimates with a model that assumes that the incidence of cancer in a region can be estimated from the number of cancer deaths observed in that region, and from the observed incidence/mortality ratio in a region with similar characteristics ^4^. In the country, there are currently five RCBP (Cali, Pasto, Bucaramanga, Manizales and Barranquilla); these cover 12% of the population.

The national estimate developed by the International Agency for Research on Cancer - IARC ^1^ is based on these two methods; but it does not offer, at province level, the disaggregation that is required for the country ^5^. Due to regional variations in the country, three studies have been carried out by the National Cancer Institute (INC, for its initials in Spanish) under this perspective: the magnitude of the disease, incidence and mortality at province level ^6^ ^-^ ^8^, information that is useful for planning human resource training, acquisition of equipment and provision of cancer prevention, detection, diagnosis and treatment services ^9^. In addition to the estimates generated by IARC, cancer information at national level also comes from other sources that do not match each other. On the one hand, there are the INC estimates based on the data from the RCBP in Cali, Manizales, Pasto and Bucaramanga; and the national system of vital statistics. The data used from RCBP comply with international quality standards endorsed by the IARC ^10^. The vital statistics system provides information on mortality, with a coverage according to data from the World Health Organization of 98.5% for 2009 ^11^, and a quality of 92.8% according to the analysis of the certification of mortality in Colombia, which was carried out for the 2002-2006 period ^12^. On the other hand, it has been necessary to have information on the quality of cancer care, which is why passive (often administrative) records on patient care have been created. From these administrative records, reports ^13^ ^,^ ^14^ which are more updated than those from RCBP have been made, but with incidence and mortality data with large differences and low reliability ^15^ ^,^ ^16^. 

The objective of this article is to present estimates of cancer incidence and mortality observed in Colombia (8), for the first five cancers in men and women (prostate, stomach, breast, cervix and colon-rectum) in the provinces, during the 2007-2011 period.

## Materials and Methods

The geographic ordering of the country is defined by regions (two or more provinces), provinces (set of several municipalities), special districts, municipalities and metropolitan areas (two or more municipalities). Based on this distribution, there were estimates made for cancer discriminated by sex, for 25 locations, in 27 provinces, the Capital District and a region that grouped the provinces of Amazonas, Guainía, Guaviare, Vaupés and Vichada. Incidence information included four RCBP, with information from 2003-2007: Cali, Metropolitan Area of ​​Bucaramanga, Manizales and Pasto. The mortality information was obtained from the official mortality databases of DANE for the period 2003-2011. The population information for the same period was obtained from the national and province estimates and projections disaggregated by sex, area and five-year age groups of DANE ^17^.

The incidence/mortality ratio was calculated with the groups by cancer location, based on the tenth edition of the International Classification of Diseases. This methodology is available in the base publication of this manuscript ^8^. For this article, we analyzed five locations corresponding to stomach (C16), colon, rectum and anus (C18-21), female breast (C50), cervix (C53), and prostate (C61) in the provinces. The complete information for the country is presented in Tables S1 and S2. 

### Mortality

Cancer mortality information required some quality adjustments in which deaths of non-residents in Colombia, deaths without sex or age information, and those certified by persons other than a physician were excluded ^8^. Deaths due to other ill-defined causes were not redistributed, nor were cancer cases or deaths from ill-defined sites, with the exception of uterine cancer of unspecified site (C55), which was redistributed among deaths from uterine cancer of specified site (C53-C54), as it is recommended by the standard methodology ^18^. The information of the province of residence was imputed by the province of occurrence of death, in those cases in which this information was not available. This allocation was not applied to the cities in which the records are located, due to the errors that could be generated when correcting this information in small geographical areas. No adjustment was made for under registration of mortality.

### Estimation of incident cases

A specific generalized linear model was used for each location, which assumes that the number of cancer cases follows a Poisson distribution and uses a logarithmic transformation as a link function. The model considered as independent variables sex and age group (0-14, 15-44, 45-54, 55-64 and ≤65 years), in addition to mortality as an offset variable, and it assumes that the incidence/mortality ratio is a constant value that is related through survival ^19^. The resulting model was the following:

*Ln*(cancer cases) - *Ln*(deaths for cancer) + B_c_ + B sex + B_2_ (age groups) + B_3_ (sex * age groups)

The model assumed that the incidence/mortality ratios would be constant in the last five years. The national estimates for each location were the result of the sum of estimated cases in each province. Readers can consult the book already published to review more details of the methodology ^8^.

### Validation of the models

The number of cases observed in each population registry was compared with that estimated from six different models in which the data from the registry or records not included as predictors are assumed to be unknown, making the estimate from the remaining records. Each of the first five models was weighed based on the square root of the population of each record; the sixth model corresponds to estimated data without considering weighing. The accurate fit of the models was not evaluated because they were all saturated.

To evaluate the statistical validity of each model, the difference between the number of cases detected by the registry and the number of cases estimated by the model was calculated; based on these differences, the sum of squared errors was calculated. Table 1 shows the model used for the estimations, which was generated from the weighted model with combined information from the Cali, Pasto, Manizales and Bucaramanga cancer registries, which obtained the lowest values of the sum of squared errors​ - SSE.

**Table 1:** Sum of the differences between the observed cancer cases and the estimated cases squared (sum of squared errors - SSE).

SSE according to the RPC included in the model	Men	Women	Total
SSE RPCC	229,603	230,699	460,302
SSE RPCC, RPCB	113,099	200,271	313,370
SSE RPCC, RPCP	193,892	182,835	376,727
SSE RPCC, RPCM	190,927	173,383	364,311
SSE RPCC, RPCP, RPCM, RPCB	113,296	142,270	255,566
SSE RPCC, RPCP, RPCM, RPCB*	126,325	145,327	271,652

* Not weighted RPC: Population Registry of Cancer;

RPCC: Population Registry of Cancer, Cali;

RPCB: Population Registry of Cancer, Bucaramanga;

RPCP: Population Registry of Cancer, Pasto;

RPCM: Population Registry of Cancer, Manizales.

Crude rates (CR) were calculated for cancer incidence and mortality per 100,000 person-years for each of the cancer sites, according to sex and provinces of the country. The CR were standardised by age (ASR) using the direct method with the world reference population (SEGI population).

## Results

### Incidence of cancer

The annual national estimate of incident cases for 2007-2011 was 62,818 cases of cancer, 29,734 in men and 33,084 in women. Age-standardised cancer incidence rates (ASIR) per 100,000, in men were 151.5 and 145.6 in women (Table S1). Women had a lower incidence ratio than men in stomach cancer (M: F: 1.8: 1), and a similar incidence ratio in colorectal cancer (M: F; 1: 1).

In men, the highest ASIR were for prostate (46.5), followed by stomach (18.5) and colorectal cancer (12.2). Among women, the highest ASIR were for breast (33.8), cervix (19.3), colorectal (12.3) and stomach (10.3). The incidence of breast cancer was twice as high as cervical cancer, and it accounted for 23.0% of all cancer cases in women and 37.6% together with cervix. The five cancers (prostate, breast, cervix, stomach and colorectal) are responsible for more than 50% of new cancer cases in Colombia (Table 2). Table S1 shows the totality of locations in greater detail.

**Table 2:** Cancer incidence and mortality, five first locations, Colombia, 2007-2011.

Characteristics		Stomach	Colorectal	Breast	Cervix	Prostate
Incident cases (year)	Total	5,955	5,185	7,627	4,462	8,872
	Men	3,613	2,401	…	…	8,872
	Women	2,342	2,784	7,627	4,462	…
Rates (men)	CR	16.3	10.8	…	…	40.0
	ASR	18.5	12.2	…	…	46.5
Rates (women)	CR	10.3	12.2	33.5	19.6	…
	ASR	10.3	12.3	33.8	19.3	…
Observed deaths (year)	Total	4,537	2,544	2,226	1,861	2,416
	Men	2,767	1,168	…	…	2,416
	Women	1,770	1,376	2,226	1,861	…
Rates (Men)	CR	12.5	5.3	…	…	10.9
	ASR	14.2	6.0	…	…	12.6
Rates (Women)	CR	7.8	6.0	9.8	8.2	…
	ASR	7.8	6.1	9.9	8.2	…

CR: crude rate

ASR: age-standardised rate (per 100,000 years-person)

**Figure 1 a-e:**
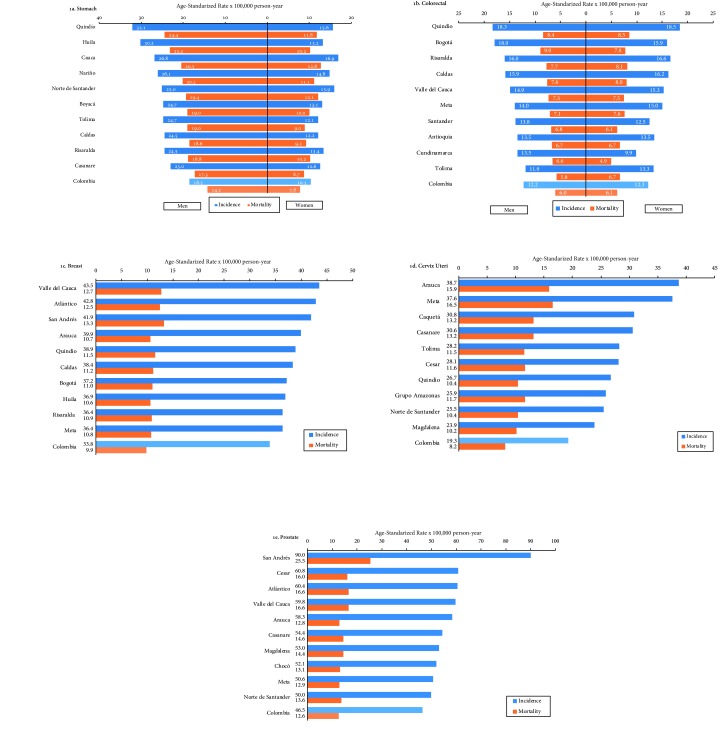
Cancer incidence and mortality, according to provinces, five main locations, Colombia, 2007-2011.

Prostate cancer corresponded to 29.8% of cancer cases among men; it presented the highest ASIR in the provinces of San Andrés and Providencia (90.0), Cesar (60.8), Atlántico (60.4) and Valle del Cauca (59.8). Similarly, breast cancer presented the highest ASIR in Valle del Cauca (43.5), Atlántico (42.8) and San Andrés y Providencia (41.9). Provinces such as Arauca (38.7), Meta (37.6) and Caquetá (30.8) had the highest incidence rates for cervical cancer ((Fig. 1a-e). 

Age-standardised cancer incidence rates for stomach in men predominated in Quindío (32.1), Huila (30.2) and Cauca (26.8); in women, they were higher in Cauca (16.9), Norte de Santander (15.9) and Quindío (15.6). In contrast, the Caribbean region presented the lowest ASIR, in a range of 3.3 -10.7 in men and 2.4 - 6.0 in women ((Table 2S). ASIR for colorectal cancer were very similar for both sexes. In men, Quindío (18.3), Bogotá (18.0) and Risaralda (16.0) predominated; in women, Quindío (18.5), Risaralda (16.6) and Caldas (16.2).

Table S2 shows the highest province rates for all cancers; 55% of cancers occurred in five provinces (Antioquia, Bogotá, Cundinamarca, Santander and Valle del Cauca). In men, the largest ASIR were in Quindío (195.5), Risaralda (182.4), Valle del Cauca (179.6) and Antioquia (173.1); in women, Quindío (193.3), Caldas (170.4), Risaralda (168.6) and Meta (167.9). The incidence of cancer in Bogotá and nine other provinces was above the national average (151.5). Men had a lower incidence ratio than women only in the provinces of Tolima (M: F; 0.9: 1) and Nariño (M: F; 0.9: 1).

### Mortality from cancer

Annual cancer deaths in men and women were 32,653; 16,081 in men and 16,572 in women, with an c (ASMR) of 82.3 in men and 73.2 in women (Table S1). The incidence / mortality ratio was 1.8 in men and 2.0 in women. Cancers in men with the highest ASMR occurred in stomach (14.2), prostate (12.6), and colorectal (6.0). In women, breast (9.9), cervix (8.2) and colorectal (6.1) (Table 2).

 Age-standardised mortality rate for provinces showed notable differences. 56.0% of the deaths occurred in the provinces of Antioquia, Bogotá, Cundinamarca, Santander and Valle del Cauca. The largest ASMR for men were found in Quindío (111.2), Risaralda (103.2), Antioquia (99.7) and Valle del Cauca (96.8); for women, Quindío (91.5), Caldas (85.5), Risaralda (91.0) and Meta (86.2) (Table S2).

In men, the provinces with the highest ASMR for stomach were in Quindío (24.4), Huila (23.2) and Cauca (20.5). In women, the highest were in Cauca (12.8), Norte de Santander (12.1) and Quindío (11.8). Prostate cancer predominated in San Andrés (25.5), Atlántico and Valle del Cauca, both with ASMR of 16.6. Mortality per breast cancer represented 13.4% among all cancer deaths in women; the highest rates presented in San Andrés (13.3), Valle del Cauca (12.7) and Atlántico (12.5). Provinces such as Meta (16.5), Arauca (15.9) and Caquetá (13.2) also had the highest mortality rates from cervical cancer. For colon-rectum, the highest mortality rates occurred in Bogotá and the Old Caldas region (Fig. 1a-e). 

## Discussion

According to these estimates, the five main locations of incidental cancers in Colombia are stomach, colorectal, breast, cervical, and prostate cancer. This is the third time that this exercise has been done for Colombia by provinces ^8^, with the incorporation of the recommendations made in past editions. For the first time, data from four cancer registries, Cali, Pasto, Manizales and the Metropolitan Area of ​​Bucaramanga are included in the estimation models, in addition to the official mortality data registered for Colombia, with 92.8% of quality in the certification. The importance of this incorporation lies in the fact that the current estimates reflect the diversity in the risk profiles for cancer incidence more for each region. A relevant factor is the quality of the information produced by the records included in the model, because they already complied with international quality standards ^2^.

The differences in the numbers incidence estimated by Globocan 2012 are related to the model selected, which includes data from cancer registries in South America and also for the use of estimated mortality in the country. Estimating the incidence/mortality ratio based on the last available year of the registry generated very volatile figures, so for these locations the information from the five-year period was used. However, for breast and prostate cancer, Globocan 2012 considered that this assumption would not be met with the relative recent introduction of screening for these pathologies, with a fluctuating result in the incidence/mortality ratios for these pathologies. This way, Globocan 2012 based the incidence/mortality ratio only on the result of the most recent year of reporting of population registries (20). On the other hand, the estimated annual figures for 2007-2011 were lower when compared with the annual figures of incidence estimated in 2002-2006, because in this study the correction for under-registration of mortality was not incorporated ^21^.

When contrasting the incidence and mortality information generated in Colombia using other sources ^13^ ^,^ ^14^, a great difference in figures is evident, both in absolute numbers and rates. Probably this discrepancy is due to the different methods used in the collection, processing and analysis of the data, sometimes resulting in less than 50% of the real number of patients present in the country ^15^.

In general, for Colombia, standardized rates of both incidence and mortality were lower than in countries such as the United States, Australia, New Zealand and countries of South America ^22^. In turn, these five types of cancer were also the main incidental cancers and the leading cause of mortality in Central and South America. However, it is highlighted that stomach cancer and cervical cancer have much higher rates than in other Latin America countries, such as Brazil, Argentina and Mexico, among others ^23^. 

The first three locations with the highest incidence (of cancer) in men were prostate, stomach and colon-rectum; and breast, cervix and colon-rectum in women. It is noteworthy that all the five main locations are characterized by having surgical treatment as a fundamental element in the comprehensive management of the disease. By geographical location, these cancers occur in five of the main provinces of Colombia, such as Antioquia, Bogotá, Cundinamarca, Santander and Valle del Cauca, which have the largest population. This situation implies in absolute terms (frequencies), the basis to make decisions about the number of health institutions needed in the different areas. In fact, this is corroborated in the panorama of oncology services enabled, mainly in surgery, and in these provinces of the country ^24^.

The incidence and mortality from stomach cancer is one of the first causes in the country, and this behavior is similar in Latin American countries such as Argentina, Brazil, Chile and Costa Rica ^23^. It is highlighted that despite the behavior, the tendency has been to decrease in recent decades ^25^ ^,^ ^26^. This decrease is attributed to the improvement in hygiene conditions and food preservation ^24^ ^,^ ^27^. In gastric cancer, the smallest difference between mortality rates versus incidence rates occurs because it is a highly fatal cancer.

Colorectal cancer corresponds to 8.3% of the incident cases in the country, and its behavior in other Latin American countries turns out to be much higher than in Colombia, even with higher rates than for stomach cancer, in countries such as the United States, Brazil and Argentina ^23^. Obesity is one of its risk factors, and its behavior in the country was of high figures of overweight according to the survey of nutritional status (ENSIN, for its Spanish acronym) from 2010. The highest prevalence of excess weight occurred in urban areas (52.5%) and in women (84.1%), which may explain the behavior of the rates.

In relation to prostate cancer, it is the first diagnosis most frequent in men, whose incidence rate (46.5) is below the rate for South America (60.1), but above the rate for Central America (28.4) ^23^ . In addition, it is the second cause of cancer mortality in Colombia, with the highest mortality rates in the coastal region and Valle del Cauca. The wide incidence ranges may be due to screening programs (prostate-specific antigen tests - PSA), to the availability of treatment services in the different regions since these provinces coincide with those with the highest percentage of Afro-Colombian population.

Something similar occurs with regional differences in breast cancer incidence rates, provinces with a large proportion of Afro-Colombian population; as well as being influenced by the availability of early detection services; and reproductive and hormonal risk associated factors, such as overweight or obesity, post-menopause, the use of menopausal hormone therapy, physical inactivity and alcohol consumption ^28^ ^,^ ^29^.

A downward trend in cervical cancer has also been found ^26^, with the highest rates in provinces of the Orinoquía region. This particular case may be associated with low access in the diagnostic process for this population. According to vaccination campaigns against HPV, coverage in 2012 was above 95% in the whole country, with the exception of Caquetá (82.2%), Vichada (74.4%) and Putumayo (86.1%) for the first phase; Guainía, Vaupés and Caquetá, among others, for the second phase ^30^. It is necessary to continue with the coverage of screening programs and to comply with vaccination coverage against human papilloma virus (HPV), whose prevalence is 16% in Latin America, in order to achieve early detection and elimination of precancerous lesions, and of HPV ^31^.

The limitations of this study include the fact that only 12% of the population in Colombia is covered by four high-quality RCBP, and the estimates were made according to the latest available information from the RCBP (quinquennium). The processes of completeness (thoroughness) and validity of the data takes longer, which generates delays in the reports of population incidence and therefore in the national estimates. 

## Conclusions

Colombia, classified as a middle income country, presented higher estimates in cancers such as breast, prostate, as well as a proportion of cancers related to infection, such as stomach and cervical cancer. Estimates from RCBP strategically located within a country constitute an effective option that is currently used by different countries such as Brazil, Colombia, Turkey and China, among others ^2^. However, RCBP as a standard strategy for cancer surveillance, burden measurement and evaluation of the impact of the disease ^32^ ^,^ ^33^ present the difficulty of having a national coverage, a situation that lies in viability and long-term sustainability ^3^ ^,^ ^34^.

Population registries of cancer and vital statistics - DANE, provide sufficient information to produce estimates at the national and province levels. It would be desirable to improve the quality of some existing cancer registries in other areas of the country, so they can be included in the estimates, and to establish some other registries, in order to expand the current coverage (12%). Only through RCBP of good quality and long trajectory can information be obtained about changes in the incidence trends.

## Supplementary material

**Table 1S:** Cancer incidence and mortality, 25 cancer categories. Colombia, 2007-2011

	Incidence Cases (year)			Rates (Men)		Rates (Women)		Observed deaths (year)			Rates (men)		Rates (women)	
Cancer category	Total	Men	Women	CR	ASR	CR	ASR	Total	Men	Women	CR	ASR	CR	AASR
All cáncer witout skin	62,818	29,734	33,084	133.9	151.5	145.3	145.6	32,653	16,081	16,572	72.4	82.3	72.8	73.2
Lips, Oral cavity and pharynx	1,494	787	707	3.5	4.0	3.1	3.1	501	295	206	1.3	1.5	0.9	0.9
Oesophagus	864	573	291	2.6	3.0	1.3	1.3	664	451	213	2.0	2.3	0.9	0.9
Stomach	5,955	3,613	2,342	16.3	18.5	10.3	10.3	4,537	2,767	1,770	12.5	14.2	7.8	7.8
Colon, rectum and anus	2,401	2,784	10.8	12.2	12.2	12.3	2,544	1,168	1,376	5.3	6.0	6.0	6.1
Liver	1,138	541	597	2.4	2.8	2.6	2.6	1,621	762	859	3.4	3.9	3.8	3.8
Gallblader	1,098	281	817	1.3	1.4	3.6	3.6	776	228	548	1.0	1.2	2.4	2.4
Pancreas	1,191	512	679	2.3	2.6	3.0	3.0	1,269	583	686	2.6	3.0	3.0	3.0
Larynx	693	575	118	2.6	3.0	0.5	0.5	377	302	75	1.4	1.6	0.3	0.3
Trachea, bronchi, lung	3,985	2,488	1,497	11.2	12.9	6.6	6.6	3,890	2,357	1,533	10.6	12.2	6.7	6.8
Skin melanoma	1,203	590	613	2.7	3.0	2.7	2.7	226	121	105	0.5	0.6	0.5	0.5
Breast	7,627	…	7,627	…	…	33.5	33.8	2,226	…	2,226	…	…	9.8	9.9
Cervix	4,462	…	4,462	…	…	19.6	19.3	1,861	…	1,861	…	…	8.2	8.2
Uterus body	771	…	771	…	…	3.4	3.5	187	…	187	…	…	0.8	0.8
Ovary and others	1,279	…	1,279	…	…	5.6	5.6	712	…	712	…	…	3.1	3.2
Prostate	8,872	8,872	…	40.0	46.5	…	…	2,416	2,416	…	10.9	12.6	…	…
Testícle	507	507	…	2.3	2.2	…	…	72	72	…	0.3	0.3	…	…
Kidney	961	537	424	2.4	2.7	1.9	1.9	379	213	166	1.0	1.1	0.7	0.7
Bladder	1,144	863	281	3.9	4.5	1.2	1.3	401	266	135	1.2	1.4	0.6	0.6
Encephalon, SCN others	1,273	702	571	3.2	3.4	2.5	2.5	952	516	436	2.3	2.5	1.9	1.9
Thyroid	2,448	247	2,201	1.1	1.3	9.7	9.4	219	56	163	0.3	0.3	0.7	0.7
Lymphoma Hodgkin	339	229	110	1.0	1.0	0.5	0.5	119	74	45	0.3	0.4	0.2	0.2
Lymphoma no Hodgkin	2,744	1,542	1,202	6.9	7.5	5.3	5.3	939	518	421	2.3	2.6	1.8	1.9
Leukemia	2,473	1,256	1,217	5.7	6.0	5.3	5.4	1,610	869	741	3.9	4.2	3.3	3.3
Other places/not especified	5,112	2,618	2,494	11.8	13.1	11.0	11.0	4,155	2,047	2,108	9.2	10.4	9.3	9.3

CR:Crude rate

ASR:age-standardised rate (per 100,000 year-person).

**Table 2S:** Cancer Incidence and mortality, Departments, Colombia, 2007-2011.

Departamet	Total	Men	Women	CR	ASR	CR	ASR	Total	Men	Women	CR	ASR	CR	ASR
Colombia	62,818	29,734	33,084	133.9	151.5	145.3	145.6	32,653	16,081	16572	72.4	82.3	72.8	73.2
Antioquia	9,781	4,606	5,175	157.4	173.1	169.0	159.9	5,419	2638	2781	90.2	99.7	90.8	86.2
Arauca	253	121	132	98.0	144.3	109.1	148.4	113	55	58	44.5	67.0	47.9	68.9
Atlántico	3,010	1,371	1,639	121.7	147.9	141.5	144.9	1,447	695	752	61.7	75.3	64.9	67.5
Bogotá	11,068	4,924	6,144	140.8	167.7	163.3	158.0	5,608	2607	3001	74.6	90.1	79.7	78.5
Bolívar	2,019	983	1,036	100.4	120.8	105.8	113.7	961	496	465	50.7	61.1	47.5	52.0
Boyacá	1,813	904	909	143.0	136.0	143.6	128.6	948	493	455	78.0	73.2	71.9	61.1
Caldas	1,860	871	989	182.2	171.2	198.5	170.4	1,008	499	509	104.4	97.5	102.2	85.5
Caquetá	447	229	218	102.6	132.0	99.6	127.4	226	117	109	52.4	68.6	49.8	66.4
Casanare	309	160	149	98.3	145.8	95.0	123.5	151	77	74	47.3	71.8	47.2	65.2
Cauca	1,521	726	795	109.5	122.2	123.2	128.6	788	394	394	59.5	66.0	61.0	63.2
Cesar	990	497	493	104.3	141.7	103.2	127.3	492	254	238	53.3	72.9	49.8	65.4
Chocó	279	145	134	61.8	99.7	56.5	78.2	128	66	62	28.1	45.0	26.2	37.1
Córdoba	1,356	687	669	87.8	105.5	86.2	97.6	661	330	331	42.2	50.6	42.6	49.0
Cundinamarca	3,157	1,546	1,611	127.0	135.0	132.0	129.9	1,649	851	798	69.9	74.2	65.4	63.7
Huila	1,451	740	711	137.9	160.3	133.6	145.9	773	410	363	76.4	88.9	68.2	74.6
La Guajira	440	214	226	54.7	83.0	56.6	72.6	201	100	101	25.5	38.8	25.3	33.5
Magdalena	1,249	634	615	105.5	133.0	104.2	122.2	633	329	304	54.8	69.5	51.5	61.6
Meta	1,206	594	612	138.5	166.9	144.3	167.9	616	313	303	73.0	88.7	71.4	86.2
Nariño	1,810	813	997	100.1	111.7	123.5	127.7	947	463	484	57.0	63.6	59.9	61.9
Norte de Santander	1,815	900	915	141.0	165.3	141.1	148.6	986	497	489	77.9	91.8	75.4	79.7
Putumayo	202	105	97	63.9	94.4	61.2	85.4	102	55	47	33.5	49.6	29.7	43.3
Quindío	1,172	557	615	207.7	195.5	220.9	193.3	616	318	298	118.6	111.2	107.1	91.5
Risaralda	1,723	828	895	184.7	182.4	189.9	168.6	958	469	489	104.6	103.2	103.8	91.0
San Andrés y Providencia	78	44	34	121.6	163.8	93.0	94.8	25	15	10	41.5	61.7	27.4	29.0
Santander	2,961	1,392	1,569	140.9	150.0	155.0	145.5	1,544	768	776	77.8	82.8	76.7	70.8
Sucre	737	360	377	88.5	100.4	95.2	103.6	379	186	193	45.7	52.0	48.7	53.4
Tolima	2,308	1,119	1,189	160.8	150.2	172.9	161.3	1,180	610	570	87.7	81.1	82.9	75.1
Valle del Cauca	7,639	3,582	4,057	170.1	179.6	181.7	167.1	4,015	1931	2084	91.7	96.8	93.3	85.1
Amazonas group *	164	82	82	50.6	82.1	54.0	83.4	79	45	34	27.8	47.4	22.4	37.3

TC: crude rate;

ASR: Age-standardised rate (por 100,000)

*Amazonas, Guainía, Guaviare, Vichada y Vaupés
