# Supplementary material for: Cancer incidence estimates and mortality for the top five cancer in Colombia, 2007-2011
Source: Colomb Med (Cali). 2018 Mar 30;49(1):16–22. doi: 10.25100/cm.v49i1.3596 (PMC6018817; doi:10.25100/cm.v49i1.3596)
Supplement: Related texts

# Research article: Estimaciones de incidencia y mortalidad para los principales cinco tipos de cáncer en Colombia, 2007-2011

## Introducción

Colombia se considera un país con una incidencia intermedia de cáncer dentro del panorama mundial, con tasas ajustadas por edad estimadas por la Agencia Internacional de Investigación en Cáncer - IARC de 175.2 casos por cada 100,000 hombres y 151.5 casos por cada 100,000 mujeres excluyendo los tumores de piel de tipo no melanoma ^1^. Para cáncer, existen métodos establecidos para medir la incidencia y mortalidad, por localización, a nivel poblacional: los registros de cáncer de base poblacional (RCBP) que recopilan información sobre la incidencia y el sistema de estadísticas vitales de los países que proporcionan datos de mortalidad ^2^. 

Lograr una cobertura nacional a través de un grupo de registros de cáncer, genera un alto costo ^3^, por esto una alternativa viable es utilizar información de unos pocos RCBP, ubicados en áreas estratégicas y los datos de mortalidad disponibles a nivel nacional para obtener estimaciones nacionales confiables, con un modelo, el cual asume que la incidencia de cáncer en una región se puede estimar a partir del número de muertes por cáncer observadas en esa región y de la relación incidencia/mortalidad observada en una región de características similares ^4^. En el país actualmente existen cinco RCBP (Cali, Pasto, Bucaramanga, Manizales y Barranquilla), y cubren el 12% de la población. 

La estimación nacional desarrollada por la Agencia Internacional de Investigación en Cáncer - IARC ^1^ se basa en estos dos métodos; pero no ofrece el nivel de desagregación departamental que se requiere para el país ^5^. Por la variación regional existente en el país se han realizado tres trabajos por parte del Instituto Nacional de Cancerología - INC bajo esta perspectiva: la magnitud de la enfermedad, la incidencia y la mortalidad a nivel departamental ^6^^-^^8^, información de utilidad para la planificación de la formación de recurso humano, la adquisición de equipos y la oferta de servicios de prevención, detección, diagnóstico y tratamiento del cáncer ^9^; además de las estimaciones generadas por la IARC, la información de cáncer a nivel nacional proviene también de otras fuentes que no resultan coincidentes. Por una parte, están las estimaciones del INC con base en los datos de los RCBP de Cali, Manizales, Pasto y Bucaramanga y el sistema nacional de estadísticas vitales. Los datos de los RCBP usados cumplen con estándares internacionales de calidad avalados por la IARC ^10^. El sistema de estadísticas vitales, proporciona información de sobre la mortalidad, con una cobertura según datos de la Organización Mundial de la Salud de 98.5% para el 2009 ^11^ y una calidad de 92.8% según análisis de la certificación de la mortalidad en Colombia realizado para 2002-2006 ^12^. 

Por otra parte, ha sido necesario disponer de información sobre la calidad de la atención del cáncer, razón por la cual se han creado registros pasivos (frecuentemente administrativos) sobre la atención del paciente. A partir de estos registros administrativos se han realizado la presentación de informes ^13^^,^^14^ más actualizados que los provenientes de los RCBP, pero con datos de incidencia y mortalidad con grandes diferencias y de baja fiabilidad ^15^^,^^16^.

El objetivo de este artículo fue presentar estimaciones de incidencia por cáncer y la mortalidad observada en Colombia ^8^, para los cinco primeros cánceres en hombres y mujeres (próstata, estómago, mama, cuello uterino y colon-recto), en el orden departamental, durante el periodo 2007-2011.

## Materiales y Métodos

El ordenamiento geográfico del país se encuentra definido en regiones (dos o más departamentos), departamentos (conjunto de varios municipios), seguido de distritos especiales, municipios y áreas metropolitanas (dos o más municipios). Con base en esta distribución se generaron cifras estimativas de cáncer discriminadas por sexo, para 25 localizaciones, en 27 departamentos, el Distrito Capital y una región que agrupó los departamentos de Amazonas, Guainía, Guaviare, Vaupés y Vichada. La información de incidencia incluyó cuatro RCBP con información de 2003-2007: Cali, Área Metropolitana de Bucaramanga, Manizales y Pasto. La información de mortalidad se obtuvo a partir de las bases de datos oficiales de mortalidad del Departamento Administrativo Nacional de Estadística - DANE para el periodo 2003-2011. La información de la población para el mismo periodo se obtuvo de las estimaciones y las proyecciones nacionales y departamentales desagregadas por sexo, área y grupos quinquenales de edad del DANE ^17^.

Se calculó la razón incidencia/mortalidad con las agrupaciones por localización del cáncer basada en la décima edición de la Clasificación Internacional de Enfermedades. Esta metodología se encuentra disponible en la publicación base de este manuscrito ^8^. Para este artículo se analizaron cinco localizaciones correspondientes a estómago (C16), colon, recto y ano (C18-21), mama de la mujer (C50), cuello uterino (C53), y próstata (C61) en el orden departamental. La información completa para el país se presenta en las Tablas S1 y S2.

### Mortalidad 

La información de mortalidad por cáncer tuvo ajustes de calidad donde se excluyeron las muertes de personas no residentes en Colombia, las muertes sin información de sexo o edad y aquellas certificadas por personas diferentes a un médico ^8^. No se redistribuyeron las muertes por otras causas mal definidas, ni los casos o las muertes por cáncer de sitios mal definidos, con excepción del cáncer de útero de sitio no especificado (C55), el cual se redistribuyó entre las muertes por cáncer de útero de sitio especificado (C53-C54), tal como lo recomienda la metodología estándar ^18^. La información del departamento de residencia se imputó por la del departamento de ocurrencia de la defunción en aquellos casos en los que esta información no estaba disponible. Esta imputación no se aplicó a las ciudades en las que se ubican los registros, debido a los errores que se podrían generar al corregir esta información en áreas geográficas pequeñas. No se hizo ajuste por subregistro de la mortalidad. 

### Estimación de casos incidentes

Se empleó un modelo lineal generalizado específico para cada localización, el cual asume que el número de casos de cáncer sigue una distribución de Poisson y utiliza una transformación logarítmica como función de enlace. El modelo consideró como variables independientes el sexo y el grupo de edad (0-14, 15-44, 45-54, 55-64 y ≤65 años), además de la mortalidad como variable *offset* y supone que la razón incidencia /mortalidad es un valor constante que se relaciona a través de la supervivencia ^19^. El modelo resultante fue el siguiente:

*Ln*(casos de cáncer) - *Ln*(muertes por cáncer) + *B*_*c*_ + *B* sexo + *B*_2_ (grupos de edad) + *B*3 (sexo*grupos de edad)

El modelo asumió que las razones de incidencia/mortalidad serían constantes en los últimos cinco años. Las estimaciones nacionales para cada localización fueron la resultante de la sumatoria de casos estimados en cada departamento. Los lectores pueden consultar el libro ya publicado para revisar más detalles de la metodología ^8^**.**

### Validación de los modelos

Se comparó el número de casos observados en cada registro poblacional con el estimado a partir de seis modelos diferentes en los que los datos del registro o registros no incluidos como predictores se suponen desconocidos, haciendo la estimación a partir de los registros restantes. Cada uno de los primeros cinco modelos se ponderó con base en la raíz cuadrada de la población de cada registro; el sexto modelo corresponde a datos estimados sin considerar ponderación. No se evaluó la bondad de ajuste de los modelos debido a que todos estaban saturados.

Para evaluar la validez estadística de cada modelo se calculó la diferencia entre el número de casos detectados por el registro y el número de casos estimados por el modelo; con base en estas diferencias se calculó la suma de errores al cuadrado. En la Tabla 1 se muestra el modelo empleado para las estimaciones, generado a partir del modelo ponderado con la información combinada de los registros de Cali, Pasto, Manizales y Bucaramanga, el cual obtuvo los valores más bajos de suma de errores al cuadrado - SEC.

**Tabla 1:** Sumatoria de las diferencias entre los casos de cáncer observados y los casos estimados elevados al cuadrado (suma de errores al cuadrado - SEC).

SEC según RPC incluido en el modelo	Hombres	Mujeres	Total
SEC RPCC	229,603	230,699	460,302
SEC RPCC, RPCB	113,099	200,271	313,370
SEC RPCC, RPCP	193,892	182,835	376,727
SEC RPCC, RPCM	190,927	173,383	364,311
SEC RPCC, RPCP, RPCM, RPCB	113,296	142,270	255,566
SEC RPCC, RPCP, RPCM, RPCB *	126,325	145,327	271,652

* No ponderado.

RPC: Registro Poblacional de Cáncer

RPCC: Registro Poblacional de Cáncer de Cali;

RPCB: Registro Poblacional de Cáncer de Bucaramanga;

RPCP: Registro Poblacional de Cáncer de Pasto;

RPCM: Registro Poblacional de Cáncer de Manizales

Se realizó el cálculo de tasas crudas (TC) para incidencia y mortalidad por cáncer por 100,000 años-persona para cada una de las localizaciones del cáncer según sexo y departamentos del país. Las TC se ajustaron por edad (TAE) mediante el método directo con la población mundial de referencia (Población SEGI). 

## Resultados

### Incidencia de cáncer

La estimación nacional anual de casos incidentes para 2007-2011 fue de 62,818 casos de cáncer, 29,734 en hombres y 33,084 en mujeres. Las tasas de incidencia de cáncer ajustadas por edad (TIAE) por 100,000, en los hombres fueron 151.5 y 145.6 en las mujeres (Tabla S1). Las mujeres tuvieron una razón de incidencia menor que los hombres en el cáncer de estómago (M:F;1,8:1), y una razón de incidencia similar en el cáncer colorrectal (M:F;1:1).

En los hombres, las TIAE más altas fueron para próstata (46.5), seguido de estómago (18.5) y cáncer colorrectal (12.2). Entre las mujeres, las TIAE más altas fueron para mama (33.8), cuello uterino (19.3), colorrectal (12.3) y estómago (10.3). La incidencia de cáncer de mama fue dos veces más alta que el cáncer cervical y representó el 23.0% de todos los casos de cáncer en mujeres y el 37.6% junto con cuello uterino. Los cinco cánceres (próstata, mama, cuello uterino, estómago y colorrectal) son responsables de más de 50% de los casos nuevos de cáncer en Colombia (Tabla 2). La Tabla S1 muestra con mayor detalle la totalidad de las localizaciones.

**Tabla 2:** Incidencia y mortalidad por cáncer, cinco primeras localizaciones, Colombia, 2007-2011.

Características		Estómago	Colon - recto		Mama	Cuello del útero	Próstata
Casos incidentes (año)	Total	5,955	5,185	7,627	4,462	8,872
	Hombre	3,613	2,401	…	…	8,872
	Mujer	2,342	2,784	7,627	4,462	…
Hombre*	TC	16.3	10.8	…	…	40.0
	TAE	18.5	12.2	…	…	46.5
Mujer*	TC	10.3	12.2	33.5	19,6	…
	TAE	10.3	12.3	33.8	19,3	…
Muertes observadas (año)	Total	4,537	2,544	2,226	1,861	2,416
	Hombre	2,767	1,168	…	…	2,416
	Mujer	1,770	1,376	2,226	1,861	…
Hombre*	TC	12.5	5.3	…	…	10.9
	TAE	14.2	6.0	…	…	12.6
Mujer*	TC	7.8	6.0	9.8	8.2	…
	TAE	7.8	6.1	9.9	8.2	…

TC, tasa cruda; TAE, tasa ajustada por edad (por 100,000 años - persona)

*tasas

El cáncer de próstata correspondió al 29.8% de los casos de cáncer entre hombres y se presentó con las TIAE más elevadas en los departamentos de San Andrés y Providencia (90.0), Cesar (60.8), Atlántico (60.4) y Valle del Cauca (59.8). De igual manera, el cáncer de mama presentó las TIAE más altas en Valle del Cauca (43.5), Atlántico (42.8) y San Andrés y Providencia (41.9). Departamentos como Arauca (38.7), Meta (37.6) y Caquetá (30.8) tuvieron las tasas de incidencia más altas por cáncer de cuello uterino ((Figura 1a-e. 

**Figura 1(a-e):** Incidencia y mortalidad por cáncer, según departamentos, cinco principales localizaciones, Colombia, 2007-2011.

Las TIAE de estómago en hombres predominaron en Quindío (32.1), Huila (30.2) y Cauca (26.8); en mujeres fue mayor en Cauca (16.9), Norte de Santander (15.9) y Quindío (15.6). En cambio la región caribe presentó sus tasas más bajas con TIAE en un rango de 3.3 -10.7 en hombres y TIAE de 2.4 - 6.0 en mujeres (Tabla S2). Las TIAE de cáncer colorrectal fueron muy similares para ambos sexos. En hombres predominaron Quindío (18.3), Bogotá (18.0) y Risaralda (16.0); en mujeres Quindío (18.5), Risaralda (16.6) y Caldas (16.2).

En la Tabla S2, se detallan las tasas departamentales más altas para todos los cánceres; el 55% de los cánceres ocurrieron en cinco departamentos (Antioquia, Bogotá, Cundinamarca, Santander y Valle del Cauca). En hombres las mayores TIAE estuvieron en Quindío (195.5), Risaralda (182.4), Valle del Cauca (179.6) y Antioquia (173.1); en mujeres se presentaron en Quindío (193.3), Caldas (170.4), Risaralda (168.6) y Meta (167.9). La incidencia de cáncer en Bogotá y nueve departamentos más estuvo por encima del promedio nacional (151.5). Los hombres tuvieron una razón menor de incidencia que en mujeres únicamente en los departamentos de Tolima (M:F;0.9:1) y Nariño (M:F;0.9:1).

## Mortalidad por cáncer

Las muertes anuales de cáncer en hombres y mujeres, fueron 32,653; 16,081 en hombres y 16,572 en mujeres, con una tasa de mortalidad ajustada por edad (TMAE) de 82.3 en hombres y de 73.2 en mujeres (Tabla S1). La razón de incidencia / mortalidad fue de 1.8 en los hombres y 2.0 en las mujeres. Los cánceres en hombres con TMAE más altas se presentaron en estómago (14.2), próstata (12.6), y colorrectal (6.0). En mujeres, mama (9.9), cuello del útero (8.2) y colorrectal (6.1) (Tabla 2).

Las TMAE departamentales mostraron diferencias notables. El 56.0% de las muertes ocurrieron en los departamentos de Antioquia, Bogotá, Cundinamarca, Santander y Valle del Cauca. Las mayores TMAE fueron para Quindío (111.2), Risaralda (103.2), Antioquia (99.7) y Valle del Cauca (96.8) en los hombres; Quindío (91.5), Caldas (85.5), Risaralda (91.0) y Meta (86.2) en mujeres (Tabla S2).

En estómago (hombres), los departamentos con TMAE más elevadas fueron Quindío (24.4), Huila (23.2) y Cauca (20.5). En mujeres se presentaron en Cauca (12.8), Norte de Santander (12.1) y Quindío (11.8). El cáncer de próstata predominó en San Andrés (25.5), Atlántico y Valle del Cauca ambas con TMAE de 16.6. La mortalidad por mama representó el 13.4% entre todas las muertes por cáncer en mujeres y las tasas más altas se presentaron en San Andrés (13.3), Valle del Cauca (12.7) y Atlántico (12.5). Departamentos como Meta (16.5), Arauca (15.9) y Caquetá (13.2) tuvieron también las tasas más altas de mortalidad por cáncer de cuello uterino. Para colon-recto las tasas más altas de mortalidad se presentaron en Bogotá y el viejo caldas (Figura 1a-e. 

## Discusión

Según estas estimaciones, las cinco localizaciones principales de cánceres incidentes en Colombia son cáncer de estómago, colorrectal, mamá, cuello uterino y próstata. Es la tercera vez que se hace este ejercicio para Colombia por departamentos ^8^, con la incorporación de las recomendaciones realizadas en las ediciones pasadas. Por primera vez se incluyen en los modelos de estimación datos de cuatro registros de cáncer, Cali, Pasto, Manizales y el Área Metropolitana de Bucaramanga; además de los datos oficiales de mortalidad registrada para Colombia, con un 92.8% de calidad en la certificación. La importancia de esta incorporación radica en que las estimaciones actuales reflejan más la diversidad en los perfiles de riesgo para incidencia de cáncer propios de cada región. Un factor relevante es la calidad de la información producida por los registros incluidos en el modelo, en razón a que estos ya cumplían con los estándares internacionales de calidad ^2^. 

Las diferencias en las cifras estimadas de incidencia por Globocan 2012 están relacionadas con el modelo seleccionado por ellos el cual incluye cifras de los registros de cáncer de Suramérica y también por el uso de mortalidad estimada para el país. Estimar la razón incidencia/mortalidad con base en el último año disponible del registro generaba cifras muy volátiles, por lo que para estas localizaciones se utilizó la información del quinquenio. Sin embargo, para cáncer de mama y próstata Globocan 2012 consideró que este supuesto no se estaría cumpliendo con la relativa reciente introducción de tamización para estas patologías, con un resultado fluctuante en las razones de incidencia/mortalidad para estas patologías. De este modo, Globocan 2012 basó la razón incidencia/mortalidad solamente en el resultado del año más reciente de reporte de los registros poblacionales ^20^. Por otra parte, las cifras anuales estimadas para 2007-2011 fueron menores al compararlas con las cifras anuales de incidencia estimada en 2002-2006 porque en este trabajo no se incorporó la corrección por subregistro de la mortalidad ^21^.

Al contrastar la información de incidencia y mortalidad generada en Colombia con otras fuentes ^13^^,^^14^, se resalta la gran diferencia en cifras, tanto números absolutos como tasas. Probablemente esta discrepancia se da por los distintos métodos utilizados en cuanto a la recolección, procesamiento y análisis de los datos, resultando a veces en menos del 50% del número real número real de pacientes presentes en el país ^15^.

En general para Colombia las tasas estandarizadas tanto de incidencia como de mortalidad fueron más bajas que la de países como Estados Unidos, Australia y Nueva Zelanda y Suramérica ^22^. A su vez estos cinco tipos de cáncer fueron también los principales cánceres incidentes y primera causa de mortalidad en Centro y Suramérica. Sin embargo, se resalta que cáncer de estómago y de cuello uterino tienen tasas mucho más altas que otros países de Latinoamérica como Brasil, Argentina y México entre otros ^23^.

Las tres primeras localizaciones de más alta incidencia en hombres fueron próstata, estómago y colon-recto; mama, cuello del útero y colon-recto en mujeres. Es de resaltar que en total las cinco localizaciones principales se caracterizan por tener tratamiento quirúrgico como un elemento fundamental en el manejo integral de la enfermedad. Por localización geográfica estos cánceres ocurren en cinco de los principales departamentos de Colombia como son Antioquia, Bogotá, Cundinamarca, Santander y Valle del Cauca que son los de mayor población. Esta situación implica en términos absolutos (frecuencias), la base para tomar decisiones sobre el número de instituciones de salud necesarias en las diferentes áreas. De hecho, esto se corrobora en el panorama de servicios oncológicos habilitados, principalmente en cirugía, y en estos departamentos del país ^24^**.**

La incidencia y mortalidad por cáncer de estómago es de las primeras causas en el país y este comportamiento resulta similar en países de Latinoamérica como Argentina, Brasil, Chile y Costa Rica ^23^. Se resalta que a pesar del comportamiento, la tendencia ha sido al descenso en las últimas décadas ^25^^,^^26^. Este descenso se atribuye a la mejora en las condiciones de higiene y conservación de los alimentos ^24^^,^^27^. En el cáncer gástrico la menor diferencia entre las tasas de mortalidad versus tasas de incidencia ocurre por tratarse de un cáncer altamente fatal. 

El cáncer colorrectal corresponde al 8.3% de los casos incidentes en el país y su comportamiento en otros países de Latinoamérica resulta ser mucho más alto que en Colombia, incluso con tasas mayores que para cáncer de estómago como son los países de Estados Unidos, Brasil y Argentina ^23^. La obesidad es uno de sus factores de riesgo y su comportamiento en el país fue de altas cifras de sobrepeso según la encuesta de situación nutricional (ENSIN) de 2010. La mayor prevalencia de exceso de peso se presentó en el área urbana (52.5%) y en mujeres (84.1%), lo cual puede explicar el comportamiento de las tasas.

En relación con el cáncer de próstata, es el primer diagnóstico más frecuente en hombres, cuya tasa de incidencia (46.5) se encuentra por debajo de la tasa para Suramérica (60.1) y por encima de la tasa para Centroamérica (28.4) ^23^. Además, es la segunda causa de mortalidad por cáncer en Colombia, con las mayores tasas de mortalidad en la región de la costa y en el Valle del Cauca. Los rangos tan amplios en la incidencia pueden deberse a los programas de tamizaje (pruebas de antígeno prostático específico - PSA), a la disponibilidad de los servicios de tratamiento en las distintas regiones y a que en estos departamentos coinciden con los de mayor porcentaje de población de raza negra en Colombia. 

Algo similar ocurre con las diferencias regionales en las tasas de incidencia por cáncer de mama, departamentos con gran proporción de población de raza negra, además de estar influidas por la disponibilidad de los servicios de detección temprana, así como los factores de riesgo reproductivos y hormonales asociados, como sobrepeso u obesidad, posmenopausia, el uso de la terapia hormonal menopáusica, inactividad física y consumo de alcohol ^28^^,^^29^.

En el cáncer de cuello uterino también con tendencia al descenso ^26^ se encuentra con las mayores tasas en departamentos de la Orinoquía. Este caso en particular puede estar asociado al bajo acceso en el proceso diagnóstico para esta población. Según las jornadas de vacunación contra el VPH, la cobertura en 2012 estuvo por encima de 95% en todo el país, con excepción de Caquetá (82.2%), Vichada (74.4%) y Putumayo (86.1%) para la primera fase; Guainía, Vaupés y Caquetá entre otros para la segunda fase ^30^**.** Es necesario continuar con la cobertura de programas de tamizaje y cumplir con las coberturas de vacunación contra el virus de papiloma humano (VPH), cuya prevalencia es del 16% en América Latina, para lograr la detección temprana y eliminación de las lesiones precancerosas, y del VPH ^31^. 

Como limitaciones del estudio está el hecho de que solamente el 12% de la población en Colombia está cubierta por cuatro RCBP de alta calidad y las estimaciones se realizaron según la última información de los RCBP (quinquenio) disponible. Los procesos de exhaustividad y validez de los datos conllevan mayor tiempo, lo cual genera retrasos en los informes de incidencia poblacional y por tanto en las estimaciones nacionales. 

## Conclusiones 

Colombia clasificado como un país de medianos ingresos presentó mayores estimaciones en cánceres como mama, próstata, además de una proporción de cánceres relacionados con la infección como son el cáncer de estómago y de cuello uterino. Las estimaciones a partir de RCBP estratégicamente ubicados dentro de un país es una opción efectiva que actualmente se usa por distintos países como Brasil, Colombia, Turquía y China entre otros ^2^. Ahora bien, los RCBP como estrategia estándar de vigilancia del cáncer, medición de la carga y evaluación del impacto de la enfermedad ^32^^,^^33^ presentan la dificultad de tener una cobertura nacional, situación que radica en la viabilidad y sostenimiento a largo plazo ^3^^,^^34^.

Los registros poblacionales de cáncer y las estadísticas vitales - DANE proveen información suficiente para producir estimaciones en el orden nacional y departamental. Sería **deseable** mejorar la calidad de algunos registros de cáncer existentes en otras áreas del país para ser incluidos en las estimaciones e instaurar algunos registros más con el fin de ampliar la cobertura actual (12%). Solamente a través de los RCBP de buena calidad y de larga trayectoria podrá tenerse información sobre el cambio en las tendencias de incidencia.

## Anexos

**Tabla 1S:** Incidencia y mortalidad por cáncer, 25 categorías de cáncer, Colombia, 2007-2011

	Casos incidentes (año)			Tasas (hombre)		Tasas (mujer)		Muertes observadas (año)			Tasas (hombre)		Tasas (mujer)	
Categoría de cáncer	Total	Hombre	Mujer	TC	TAE	TC	TAE	Total	Hombre	Mujer	TC	TAE	TC	TAE
Todos los cánceres excepto piel	62,818	29,734	33,084	133.9	151.5	145.3	145.6	32,653	16,081	16,572	72.4	82.3	72.8	73.2
Labio, cavidad oral y faringe	1,494	787	707	3.5	4.0	3.1	3.1	501	295	206	1.3	1.5	0.9	0.9
Esófago	864	573	291	2.6	3.0	1.3	1.3	664	451	213	2.0	2.3	0.9	0.9
Estómago	5,955	3,613	2,342	16.3	18.5	10.3	10.3	4,537	2,767	1,770	12.5	14.2	7.8	7.8
Colon, recto y ano	5,185	2,401	2,784	10.8	12.2	12.2	12.3	2,544	1,168	1,376	5.3	6.0	6.0	6.1
Hígado	1,138	541	597	2.4	2.8	2.6	2.6	1,621	762	859	3.4	3.9	3.8	3.8
Vesícula biliar	1,098	281	817	1.3	1.4	3.6	3.6	776	228	548	1.0	1.2	2.4	2.4
Páncreas	1,191	512	679	2.3	2.6	3.0	3.0	1,269	583	686	2.6	3.0	3.0	3.0
Laringe	693	575	118	2.6	3.0	0.5	0.5	377	302	75	1.4	1.6	0.3	0.3
Tráquea, bronquios y pulmón	3,985	2,488	1,497	11.2	12.9	6.6	6.6	3,890	2,357	1,533	10.6	12.2	6.7	6.8
Melanoma de la piel	1,203	590	613	2.7	3.0	2.7	2.7	226	121	105	0.5	0.6	0.5	0.5
Mama	7,627	…	7,627	…	…	33.5	33.8	2,226	…	2,226	…	…	9.8	9.9
Cuello del útero	4,462	…	4,462	…	…	19.6	19.3	1,861	…	1,861	…	…	8.2	8.2
Cuerpo del útero	771	…	771	…	…	3.4	3.5	187	…	187	…	…	0.8	0.8
Ovario y otros anexos	1,279	…	1,279	…	…	5.6	5.6	712	…	712	…	…	3.1	3.2
Próstata	8,872	8,872	…	40.0	46.5	…	…	2,416	2,416	…	10.9	12.6	…	…
Testículo	507	507	…	2.3	2.2	…	…	72	72	…	0.3	0.3	…	…
Riñón	961	537	424	2.4	2.7	1.9	1.9	379	213	166	1.0	1.1	0.7	0.7
Vejiga	1,144	863	281	3.9	4.5	1.2	1.3	401	266	135	1.2	1.4	0.6	0.6
Encéfalo y otros del SNC	1,273	702	571	3.2	3.4	2.5	2.5	952	516	436	2.3	2.5	1.9	1.9
Tiroides	2,448	247	2,201	1.1	1.3	9.7	9.4	219	56	163	0.3	0.3	0.7	0.7
Linfoma Hodgkin	339	229	110	1.0	1.0	0.5	0.5	119	74	45	0.3	0.4	0.2	0.2
Linfoma no Hodgkin	2,744	1,542	1,202	6.9	7.5	5.3	5.3	939	518	421	2.3	2.6	1.8	1.9
Leucemia	2,473	1,256	1,217	5.7	6.0	5.3	5.4	1,610	869	741	3.9	4.2	3.3	3.3
Otros sitios y los no especificados	5,112	2,618	2,494	11.8	13.1	11.0	11.0	4,155	2,047	2,108	9.2	10.4	9.3	9.3

TC: tasa cruda

TAE: tasa ajustada por edad (por 100,000).

**Tabla 2S:** Incidencia y mortalidad por cáncer, por Departamentos, Colombia, 2007-2011.

Departamento	Total	Hombre	Mujer	TC	TAE	TC	TAE	Total	Hombre	Mujer	TC	TAE	TC	TAE
Colombia	62,818	29,734	33,084	133.9	151.5	145.3	145.6	32,653	16,081	16572	72.4	82.3	72.8	73.2
Antioquia	9,781	4,606	5,175	157.4	173.1	169.0	159.9	5,419	2638	2781	90.2	99.7	90.8	86.2
Arauca	253	121	132	98.0	144.3	109.1	148.4	113	55	58	44.5	67.0	47.9	68.9
Atlántico	3,010	1,371	1,639	121.7	147.9	141.5	144.9	1,447	695	752	61.7	75.3	64.9	67.5
Bogotá	11,068	4,924	6,144	140.8	167.7	163.3	158.0	5,608	2607	3001	74.6	90.1	79.7	78.5
Bolívar	2,019	983	1,036	100.4	120.8	105.8	113.7	961	496	465	50.7	61.1	47.5	52.0
Boyacá	1,813	904	909	143.0	136.0	143.6	128.6	948	493	455	78.0	73.2	71.9	61.1
Caldas	1,860	871	989	182.2	171.2	198.5	170.4	1,008	499	509	104.4	97.5	102.2	85.5
Caquetá	447	229	218	102.6	132.0	99.6	127.4	226	117	109	52.4	68.6	49.8	66.4
Casanare	309	160	149	98.3	145.8	95.0	123.5	151	77	74	47.3	71.8	47.2	65.2
Cauca	1,521	726	795	109.5	122.2	123.2	128.6	788	394	394	59.5	66.0	61.0	63.2
Cesar	990	497	493	104.3	141.7	103.2	127.3	492	254	238	53.3	72.9	49.8	65.4
Chocó	279	145	134	61.8	99.7	56.5	78.2	128	66	62	28.1	45.0	26.2	37.1
Córdoba	1,356	687	669	87.8	105.5	86.2	97.6	661	330	331	42.2	50.6	42.6	49.0
Cundinamarca	3,157	1,546	1,611	127.0	135.0	132.0	129.9	1,649	851	798	69.9	74.2	65.4	63.7
Huila	1,451	740	711	137.9	160.3	133.6	145.9	773	410	363	76.4	88.9	68.2	74.6
La Guajira	440	214	226	54.7	83.0	56.6	72.6	201	100	101	25.5	38.8	25.3	33.5
Magdalena	1,249	634	615	105.5	133.0	104.2	122.2	633	329	304	54.8	69.5	51.5	61.6
Meta	1,206	594	612	138.5	166.9	144.3	167.9	616	313	303	73.0	88.7	71.4	86.2
Nariño	1,810	813	997	100.1	111.7	123.5	127.7	947	463	484	57.0	63.6	59.9	61.9
Norte de Santander	1,815	900	915	141.0	165.3	141.1	148.6	986	497	489	77.9	91.8	75.4	79.7
Putumayo	202	105	97	63.9	94.4	61.2	85.4	102	55	47	33.5	49.6	29.7	43.3
Quindío	1,172	557	615	207.7	195.5	220.9	193.3	616	318	298	118.6	111.2	107.1	91.5
Risaralda	1,723	828	895	184.7	182.4	189.9	168.6	958	469	489	104.6	103.2	103.8	91.0
San Andrés y Providencia	78	44	34	121.6	163.8	93.0	94.8	25	15	10	41.5	61.7	27.4	29.0
Santander	2,961	1,392	1,569	140.9	150.0	155.0	145.5	1,544	768	776	77.8	82.8	76.7	70.8
Sucre	737	360	377	88.5	100.4	95.2	103.6	379	186	193	45.7	52.0	48.7	53.4
Tolima	2,308	1,119	1,189	160.8	150.2	172.9	161.3	1,180	610	570	87.7	81.1	82.9	75.1
Valle del Cauca	7,639	3,582	4,057	170.1	179.6	181.7	167.1	4,015	1931	2084	91.7	96.8	93.3	85.1
Grupo Amazonas*	164	82	82	50.6	82.1	54.0	83.4	79	45	34	27.8	47.4	22.4	37.3

TC: tasa cruda;

TAE: tasa ajustada por edad (por 100,000)

*Amazonas, Guainía, Guaviare, Vichada y Vaupés
